# Diazepam-Associated Hypotension Contributing to Recurrent Syncope in an Older Adult

**DOI:** 10.7759/cureus.114002

**Published:** 2026-08-05

**Authors:** Manar Shalak, Nafiisah Rajabalee, Harsha Sai Sreemantula,

**Affiliations:** 1 Geriatrics and Palliative Care, West Virginia University Hospitals, Morgantown, USA; 2 Geriatrics, Temple University Hospital, Philadelphia, USA; 3 Internal Medicine, St. Francis Medical Center, Trenton, USA

**Keywords:** benzodiazepines, geriatrics, orthostatic hypotension, polypharmacy, syncope

## Abstract

Syncope in older adults is often multifactorial, with polypharmacy being a common and potentially modifiable factor. Benzodiazepines are prescribed for anxiety and insomnia in this population despite being associated with adverse effects, including falls, cognitive impairment, and hypotension.

We present the case of a 68-year-old female patient with multimorbidity and recurrent unexplained syncope despite extensive cardiologic and neurologic evaluations. She was on diazepam therapy for anxiety as part of a complex medication regimen. A temporal relationship was observed between diazepam administration and episodes of hypotension, with systolic blood pressure dropping as low as 70 mmHg approximately three to four hours after dosing. Following stepwise dose reduction of diazepam, the frequency of syncopal episodes decreased over a four-month period, with no alternative single factor fully explaining the improvement.

This case highlights the importance of medication review in older adults presenting with syncope. Benzodiazepines may contribute to clinically significant hypotension, particularly in the context of polypharmacy, and dose reduction or deprescribing may lead to clinical improvement.

## Introduction

Syncope, defined as a transient loss of consciousness secondary to global cerebral hypoperfusion followed by spontaneous and complete recovery, represents a highly prevalent clinical challenge in older adults [1]. In this population, etiology is frequently multifactorial, driven by an interplay of age-related physiological changes, multimorbidity, and polypharmacy [2]. Commonly defined as the concurrent use of five or more medications, polypharmacy is a principal risk factor for orthostatic and drug-induced hypotension [3]. Orthostatic hypotension itself, frequently precipitated or aggravated by vasoactive and centrally acting medications, is a significant and potentially reversible contributor to syncopal events, with a reported prevalence ranging from 5% to 33% in geriatric cohorts [2,4].

Despite well-documented associations with adverse outcomes such as falls, cognitive impairment, and excessive sedation, benzodiazepine prescribing rates increase with advancing age, reaching up to 8.7% among adults aged 65 to 80 years [2,5,6]. Although the exact pathophysiological mechanisms underlying benzodiazepine-induced hypotension remain incompletely understood, proposed pathways include central suppression of sympathetic outflow, direct myocardial depression, and peripheral vasodilation [7]. Here, we describe a complex clinical case highlighting benzodiazepine-mediated hypotension and the challenges of managing polypharmacy in an elderly patient presenting with recurrent syncopal episodes.

## Case presentation

**Figure 1:**
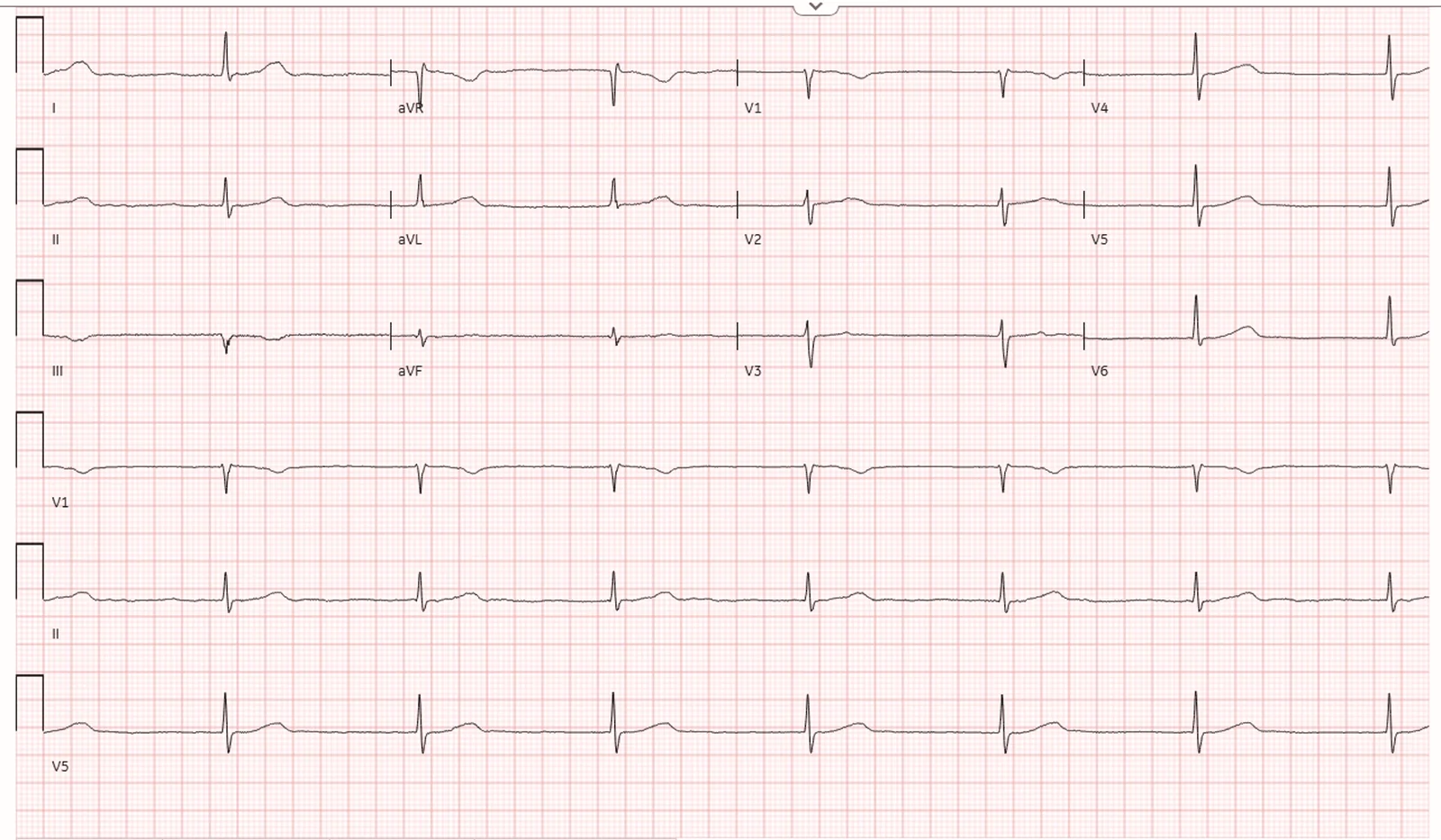
Initial 12-lead EKG on presentation The EKG demonstrates junctional bradycardia prior to pacemaker implantation at the beginning of her clinical course, although a different etiology was pursued later.

We present the case of a 68-year-old female patient with a history including paroxysmal atrial fibrillation on anticoagulation and rate control, chronic hyponatremia, hypertension, generalized anxiety disorder, and ischemic necrosis of the colon, status post subtotal colectomy with ileostomy (October 2023), who was evaluated in the geriatric outpatient clinic for recurrent syncope. Over the past year, the patient experienced recurrent, unwitnessed syncopal episodes occurring approximately once per month. The duration of these events was unknown, but their frequency resulted in multiple hospitalizations and extensive cardiologic and neurologic evaluations. These episodes were initially thought to be related to her cardiac background, including junctional bradycardia treated with a permanent pacemaker and paroxysmal atrial fibrillation treated with a beta-blocker and apixaban. Figure 1 shows an EKG demonstrating junctional bradycardia at the beginning of her clinical course before she had the pacemaker. Initial laboratory testing, including a complete blood count, comprehensive metabolic panel, blood glucose, and thyroid studies, was unremarkable except for a baseline chronic, stable normocytic anemia (hemoglobin 10.2 g/dL, mean corpuscular volume (MCV) 83 fL) with iron studies indicative of anemia of chronic disease. Extensive diagnostic evaluations, comprising EEG, brain imaging, echocardiogram, and nuclear stress testing, yielded unrevealing results for a clear cause of her symptoms.

Her medication regimen was notable for polypharmacy and included amiodarone 200 mg twice daily, apixaban 5 mg twice daily, clopidogrel 75 mg once daily, metoprolol succinate 100 mg once daily, lisinopril 5 mg once daily, rosuvastatin 20 mg once daily, vitamin D3 1000 International Units once daily, fluoxetine 10 mg once daily, sodium chloride tablets 1,000 mg three times daily, tamsulosin 0.4 mg once daily, and diazepam 5 mg twice daily.

By the time of her geriatric evaluation, one year following symptom onset, the frequency of her syncopal episodes had escalated to approximately two to three times per month. Initially occurring without warning, more recent episodes were preceded by dizziness and generalized weakness. Her medication review raised concern for a potential medication-related contribution, not only from her cardiac medications and tamsulosin but more particularly from diazepam, which she had been taking since 2021 for anxiety. A temporal relationship became apparent between diazepam administration and hypotensive episodes. Home and clinic readings showed systolic blood pressures as low as 70 mmHg at around three to four hours post dose of diazepam. Clinic blood pressures of 84/56 mmHg and 73/59 mmHg were recorded with a stable pulse of 71 bpm, consistent with symptomatic hypotension. These episodes often occurred during morning physical therapy sessions and impaired her ability to participate. Blood pressure variability was also noted, with intermittent systolic elevations up to 180 mmHg. Her volume status was unchanged and remained euvolemic.

Following a shared decision-making discussion, the dose of diazepam was reduced to 3 mg twice daily, and remote home blood pressure monitoring was started. Over the subsequent four months, the frequency of her syncopal episodes decreased significantly, with only a single event occurring, notably triggered by standing while traveling to a morning appointment, which required emergency evaluation. She improved after receiving one liter of intravenous fluids.

During the same period, the patient had two emergency department visits for worsening anxiety following the inadvertent discontinuation of fluoxetine during a rehabilitation stay. Consequently, fluoxetine was restarted, and alprazolam (1 mg nightly) was introduced as needed. Diazepam was subsequently tapered further to two mg twice daily. Over the following five to nine months, her home blood pressure readings demonstrated a sustained reduction (systolic decrease of 15-20 mmHg; diastolic decrease of ~10 mmHg), and she experienced only a single syncopal episode. No alternative etiologies or changes accounted for this marked clinical improvement. A detailed timeline of diazepam dose adjustments and the corresponding frequency of syncope is provided in Table 1.

**Table 1:** Timeline of diazepam dose reduction, BP trends, and syncopal events This table represents the time association of the patient's syncope with diazepam dose over a nine-month period. BID: twice a day; BP: blood pressure; PRN: as needed

Time Point	Diazepam Dose	BP Findings	Syncopal Events	Clinical Notes
Baseline (Geriatric evaluation)	5 mg BID	Symptomatic hypotension three to four hours post dose; systolic BP 73–84 mmHg (clinic BP 73/59 and 84/56 mmHg); intermittent systolic elevations up to 180 mmHg	2–3 episodes/month	Syncopal episodes increasingly preceded by dizziness and weakness; impaired participation in physical therapy
Month 0	Reduced to 3 mg BID	Home BP monitoring initiated	—	Concern for diazepam-associated hypotension identified through medication review
Months 1–2	3 mg BID	Improvement in BP stability	One episode requiring ED visit over four months	Significant reduction in syncopal frequency despite no other clear explanatory factor
Month 3	Further reduced to 2 mg BID	Average systolic BP decreased by approximately 15–20 mmHg and diastolic BP by ~10 mmHg compared with baseline variability	—	Fluoxetine inadvertently discontinued during rehabilitation stay; anxiety worsened, requiring two ED visits
Month 4	2 mg BID (with intermittent alprazolam 1 mg PRN and resumed fluoxetine)	Lower and more stable average BP maintained	One syncopal episode over four months	Continued clinical improvement despite intermittent benzodiazepine use
Months 5-9	2 mg BID (with intermittent alprazolam 1 mg PRN and resumed fluoxetine)	Lower and more stable average BP maintained	0 syncopal episodes	

## Discussion

This case highlights the difficulty in assessing syncope in the elderly, who often have multiple contributing factors. The patient’s symptoms were initially attributed to cardiac comorbidities, but episodes continued despite a negative workup, leading to an in-depth assessment of possible causes and a more detailed review of her medication regimen. Polypharmacy is likely a major factor. The combination of metoprolol succinate, lisinopril, and tamsulosin may have increased her risk of hypotension, with diazepam as an additional factor.

Determining the primary etiology of hypotension required navigating several confounding pharmacotherapeutic and clinical factors. While selective serotonin reuptake inhibitors like fluoxetine can precipitate orthostatic hypotension, the distinct temporal alignment between diazepam administration and the patient's hypotensive episodes, along with prompt clinical improvement following dose reduction, strongly points to a benzodiazepine-mediated mechanism. Furthermore, the transient hypertension observed upon abrupt fluoxetine cessation was characteristic of antidepressant withdrawal rather than baseline orthostasis, justifying the clinical decision to reinstate fluoxetine given her history of chronic anxiety. The progressive reduction in the frequency of orthostatic hypotension and syncope following stepwise tapering of diazepam strongly suggests a dose-dependent effect. While both alprazolam and diazepam can theoretically induce hypotension, the patient's sustained improvement despite intermittent alprazolam use further supports a dose-response relationship, though a broader class effect cannot be entirely ruled out.

Hypovolemia was also evaluated as a potential contributor. Although the patient demonstrated clinical improvement following the administration of intravenous fluids, baseline physical examination findings and laboratory evaluations lacked evidence of volume depletion, indicating that hypovolemia was not the primary driver of her presentation. Mild, stable chronic anemia presented an additional potential confounder, but it did not account for the acute, dose-dependent hemodynamic changes.

A central challenge in managing this case was the inability to fully de-escalate her baseline antihypertensive and vasoactive regimen due to competing clinical priorities. Lisinopril could not be safely discontinued due to severe blood pressure variability marked by systolic spikes up to 180 mmHg. Additionally, metoprolol succinate was clinically required to maintain rate and symptom control for her paroxysmal atrial fibrillation, while a brief trial of holding tamsulosin resulted in immediate urinary retention that necessitated its rapid resumption. Ultimately, despite these necessary background medications and minor confounding variables, the close temporal relationship between the reduction of the patient's high daily dose of diazepam and her subsequent clinical recovery confirms benzodiazepine-induced hypotension as the primary clinical driver. 

The clinical impact extended beyond episodic syncope. Recurrent events interfered with participation in physical therapy and increased healthcare utilization, placing additional strain on both the patient and her caregiver. This shows the importance of considering functional outcomes when evaluating syncope in older adults. A structured medication review is a key step in evaluating syncope and orthostatic hypotension in this population, particularly in the setting of polypharmacy [7]. Older adults exhibit increased sensitivity to benzodiazepines and reduced clearance of long-acting agents [8]. Moreover, diazepam, being highly lipophilic, has an increased volume of distribution and prolonged elimination in this population, which may amplify its hemodynamic effects [9]. The mechanism by which benzodiazepines lower blood pressure is not fully understood but likely involves reduced sympathetic tone, possible negative inotropic effects, and skeletal muscle relaxation, leading to venous pooling [10]. In this case, the timing of hypotension, occurring several hours after dosing, suggests a pharmacodynamic relationship consistent with peak drug effect.

The case also illustrates real-world challenges in deprescribing. Complete discontinuation of benzodiazepines was limited by underlying anxiety, long-term use, and fragmented care across healthcare settings, including medication changes during rehabilitation. These challenges are common in geriatric practice and highlight the importance of careful medication reconciliation during transitions. Current geriatric prescribing guidelines recommend avoiding benzodiazepines when possible due to their association with falls, cognitive impairment, and adverse events [11]. In patients presenting with unexplained syncope, especially in the context of polypharmacy, medications with hypotensive or sedative effects should be carefully reviewed and adjusted when feasible.

## Conclusions

This case highlights once again that syncope in older adults is often multifactorial, with medications representing an important and potentially modifiable contributor. In this patient, the temporal association between diazepam use, documented hypotension, and improvement following dose reduction suggests a medication-related component within a complex clinical context.

Even in patients with significant cardiovascular comorbidities, a structured medication review can reveal contributors that may otherwise be overlooked. Benzodiazepines, particularly in the setting of polypharmacy, should be carefully evaluated given their potential to exacerbate hypotension and increase the risk of syncope and falls. This case underscores the value of a comprehensive geriatric approach, where attention to pharmacologic burden, functional impact, and care transitions can lead to improvements in patient outcomes.
